# Diagnostic challenge in veterinary pathology: Tri-cavitary effusion in a cat with systemic pyogranulomatous inflammation

**DOI:** 10.1177/03009858241226648

**Published:** 2024-01-21

**Authors:** Sai Fingerhood, Pradeep Neupane, Edward B. Breitschwerdt, Eunju April Choi

**Affiliations:** 1 University of Surrey, Guildford, UK; 2 North Caroline State University, Raleigh, NC; 3 University of California, Davis, Davis, CA

## Clinical History and Laboratory Results

An approximately 2-month-old, domestic shorthair cat presented to the University of California, Davis Veterinary Medical Teaching Hospital for lethargy, hyporexia, and increased respiratory rate and effort of a few days duration. Clinically, there was tri-cavitary effusion; the pleural effusion, interpreted as a borderline modified transudate, had a nucleated cell count of 130/µl (43% neutrophils, 29% small mononuclear cells, 28% large mononuclear cells), a total protein of 2.5 g/dl, and frequent erythrophagia. Complete blood count abnormalities included an inflammatory leukogram with a left shift (4680 band/µl, 120 metamyelocytes/µl), toxic neutrophils, and a nonregenerative anemia (17.2% hematocrit [30%–50%]). Serum biochemical abnormalities included hypoglobulinemia (2.0 g/dl [2.8–5.4]) with a low-normal albumin (2.4 g/dl [2.2–4.6]), hyponatremia (133 mmol/l [151–158]), hypokalemia (3.1 mmol/l [3.6–4.9]), hypochloremia (101 mmol/l [117–126]), hypophosphatemia (3 mg/dl [3.2–6.3]), and hypoglycemia (59 mg/dl [63–118]). Pleural fluid was quantitative PCR negative for feline coronavirus (feline infectious peritonitis [FIP]), and a feline leukemia virus antigen SNAP test (IDEXX Laboratories, Westbrook, ME, USA) was negative. Echocardiography and chest radiographs did not identify congenital cardiac abnormalities. Due to a guarded prognosis, humane euthanasia was elected.

## Gross Findings

**Figure 1.**
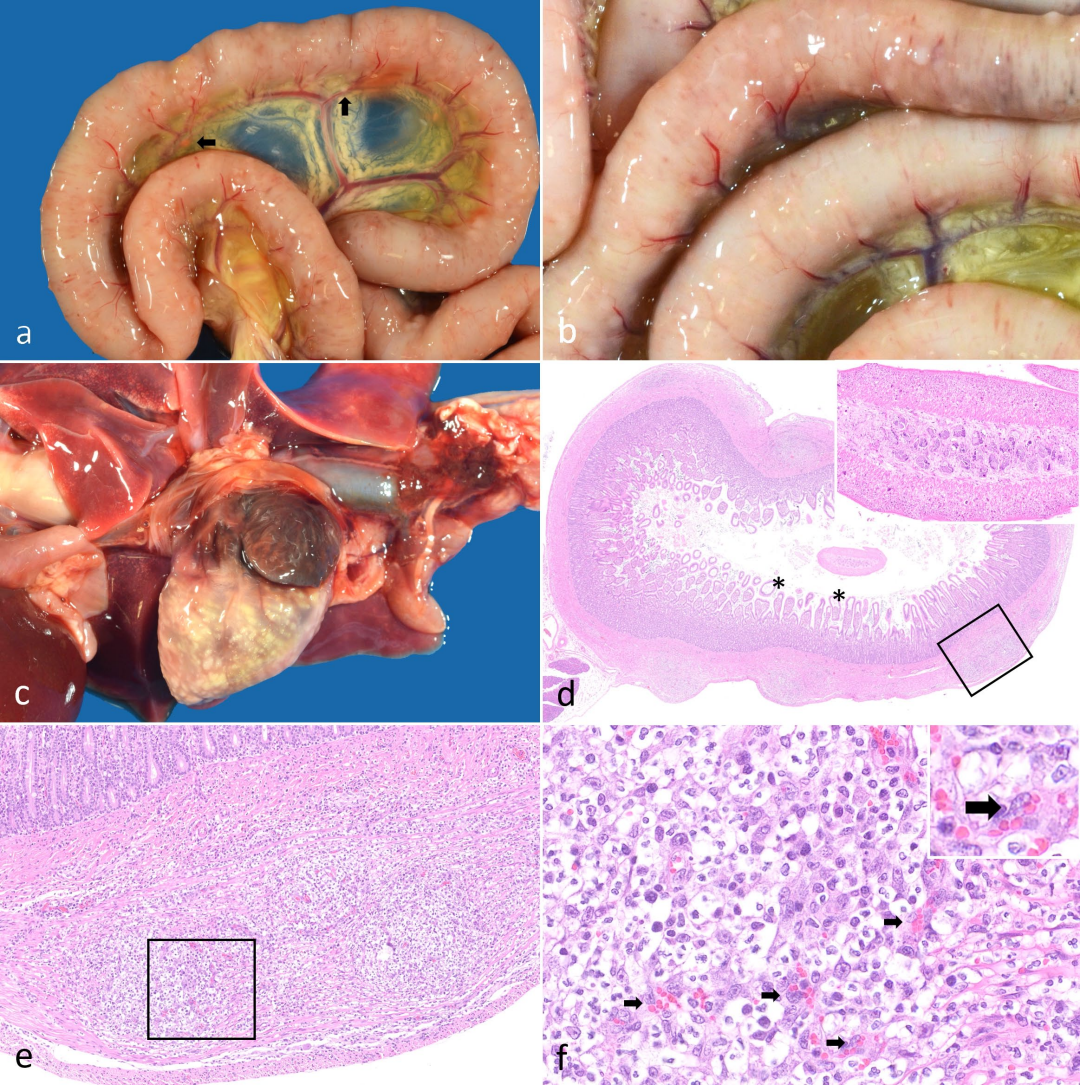
Nodular pyogranulomatous intestinal leiomyositis and myocarditis in a cat. a, b) Small intestine. The serosa of the small intestine is multifocally elevated with hundreds of pinpoint to 1- to 2-mm-diameter, firm, well-demarcated, cream-to-yellow nodules. The nodules occasionally track along the serosal vasculature and rarely track along the mesenteric vasculature (black arrows). c) Heart. The myocardium and epicardium are disrupted by nodules similar to those present in the small intestine. d) Duodenum. The outer tunica muscularis of the duodenum is multifocally disrupted with nodular accumulations of inflammatory cells. Villous lymphatics are moderately dilated (asterisks). Inset: Within the lumen is a transverse profile of a 0.75- to 1-µm-diameter cestode characterized by a parenchymous body cavity, a thin eosinophilic tegument, and numerous subtegumental oval, clear structures containing central basophilic to eosinophilic amorphous material (calcareous corpuscles). Hematoxylin and eosin (HE). e) Duodenum. Higher magnification of the boxed region in (d). The serosa is elevated by a disorganized accumulation of inflammatory cells centered around prominent small vessels, which together disrupt the architecture of the tunica muscularis. Inflammation mildly extends into the submucosa. HE. f) Duodenum. Higher magnification of the boxed region in (e). The inflammation consists of neutrophils, fibrillar eosinophilic material (fibrin), and histiocytes. Reactive small vessels are prominent within these nodules (black arrows). These small vessels are lined by hypertrophied, fusiform endothelia that have large round-to-ovoid nuclei and vesiculate chromatin with variably present, prominent nucleoli. HE.

Gross postmortem examination confirmed a mild tri-cavitary effusion, consistent with a modified transudate; the peritoneal and pleural cavities contained rare strands of fibrin. The subcutis was diffusely and markedly edematous. The serosal surfaces of all segments of the gastrointestinal tract (esophagus to colon) and the diaphragm were multifocally elevated with numerous, pinpoint to 2-mm diameter, firm, well-demarcated, cream-to-yellow nodules, which occasionally tracked along the serosal and mesenteric vasculature (Fig. 1a, b). Similar, densely packed nodules elevated the epicardial surface of both ventricles (Fig. 1c). There was cestodiasis of the jejunum, consistent with *Dipylidium caninum* (Fig. 1d).

## Microscopic Findings

The described nodules corresponded to inflammatory foci within the gastrointestinal smooth muscle (esophagus, stomach, small intestine, cecum, colon); myocardium, endocardium, and epicardium; and skeletal muscle (diaphragm, cervical), with additional microscopic foci identified within the pancreas. The architecture of these tissues was multifocally disrupted by nodular accumulations of neutrophils, fibrillar eosinophilic material (fibrin), and histiocytes, centered around reactive blood vessels with hypertrophied endothelia (Fig. 1d–f). Within intestinal and epicardial sections, the multifocal nodules elevated the serosal surfaces. Inflammation minimally extended into the submucosa and surrounding mesentery of the intestinal sections. There was mild segmental lymphatic dilation within the lamina propria of the small intestine.

## Differential Diagnoses

Given the tri-cavitary effusion and the multifocal nodular pattern of inflammation within multiple organ systems, a systemic inflammatory process with muscle tropism was suspected. Differentials for systemic inflammatory processes included infectious causes (viral, bacterial, protozoal), immune-mediated reactions (typically type III hypersensitivities), and idiosyncratic drug and toxin reactions. The main differential for pyogranulomatous inflammation in young cats is FIP. Diagnostic testing for this virus can be pursued via PCR, as was the case here, or via immunohistochemistry (IHC), the latter of which is considered more sensitive. Other considerations for infectious causes of pyogranulomatous inflammation in cats include toxoplasmosis, mycobacteriosis, and systemic fungal infections, though the former tends to be more necrotizing and the latter two are not typically myotropic. The multifocal, systemic inflammation raised concern for a primary vasculitis; however, the lack of leukocytoclasia or fibrinoid vascular necrosis decreased our suspicion of an immune-mediated reaction or specific bacterial etiologies. The large numbers of small, prominent blood vessels at the center of inflammatory nodules also raised concern for a vasoproliferative process; however, it was not possible to differentiate between a purely reactive process and one that was definitively vasoproliferative. Pyogranulomatous myositis has been attributed to infection with *Bartonella* spp., which was also considered as a differential diagnosis.^10^

## Further Investigations and Diagnosis

**Figure 2.**
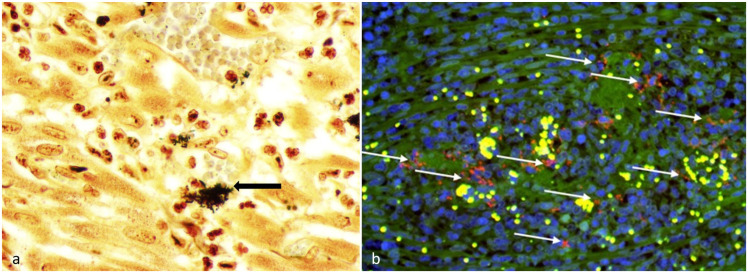
Silver impregnation of heart and indirect immunofluorescence for *Bartonella henselae* in the small intestine of a cat. a) Heart. Aggregates of argyrophilic coccobacilli are scattered within a focal region of myocardial inflammation; some of these bacteria are within vessel lumina (black arrow). Warthin-Starry. b) Small intestine. *B. henselae* immunoreactivity (shown in red, white arrows) within a focus of pyogranulomatous leiomyositis, visualized using indirect immunofluorescence. Green, autofluorescence; blue (DAPI), nucleus; yellow, erythrocytes; and red, *B. henselae.*

IHC for FIP virus and *Toxoplasma gondii* were both negative on intestinal sections. Warthin-Starry impregnation of formalin-fixed paraffin-embedded sections of myocardium revealed small aggregates of argyrophilic bacteria within a focus of cardiac inflammation (Fig. 2a). Aerobic cultures of the myocardium resulted in no growth. Given the results of the silver impregnation, testing for *Bartonella* was pursued at North Carolina State University. Using conventional and quantitative PCR targeting the *Bartonella henselae 16S-23S rRNA ITS* region, *B. henselae* DNA (100% identity, 573/573 bp) was amplified and sequenced from frozen heart and colon, using previously described methods.^8^ Indirect immunofluorescence for *B. henselae* was positive in multiple organs, with organisms identified within regions of inflammation in the intestine (Fig. 2b). Given these results, we considered the most likely cause of the pyogranulomatous gastrointestinal leiomyositis, myositis, and pancarditis to be *B. henselae*.

## Discussion

This case provides molecular and immunofluorescent evidence of *B. henselae* infection of smooth, skeletal, and cardiac muscles in a young cat. *Bartonella* species are vector-borne, highly fastidious, gram-negative, zoonotic, alpha proteobacteria that cause chronic intra-erythrocytic and vascular-endothelial infections with relapsing bacteremia in a variety of host species.^3^ The most commonly implicated vector is the cat flea (*Ctenocephalides felis*), though the bacteria has also been found in other arthropod vectors, including *Ixodes* spp. ticks.^2^ The incidental presence of cestodiasis in this cat may reflect historic flea infestation, given that *C. felis* is also the vector for *Dipylidium* spp.. The most common histopathologic lesions that have been associated with bartonellosis in cats include pyogranulomatous endocarditis, myocarditis, endomyocarditis, and systemic reactive angioendotheliomatosis.^4^ Fever, neurologic signs, lymphadenitis, and endocarditis have been associated with infections.^2^ Pre-mortem serology, PCR, and culture can be employed to support a diagnosis of bartonellosis; however, the high prevalence of subclinical or asymptomatic infections within felid populations makes establishing disease causation difficult.^1^

Cats are considered the main reservoir for *B. henselae*, resulting in human infections being colloquially referred to as “cat scratch disease,” which is characterized by erythematous skin papules and lymphadenitis with a history of cat scratch or bite.^2^ In humans, *B. henselae* has also been associated with vasculoproliferative syndromes, including peliosis hepatitis, and bacillary angiomatosis, most often reported in immunocompromised patients.^6^ Infections have more rarely been associated with inflammatory bowel disease,^7^ granulomatous hepatitis,^7^ and in one report, a mural, granulomatous duodenal mass that resolved with antibiotic treatment.^9^ In dogs, *B. henselae* has been associated with granulomatous endocarditis and pancarditis, uveitis, meningitis, encephalitis, lymphadenitis, and hepatitis.^5^

Host immunocompetency plays an important role in *B. hensleae* disease manifestations. In this case, the cat’s young age likely played a role in disease development.^1^ Bartonellosis pathogenesis involves both extraerythrocytic and intraerythrocytic stages and includes multiple putative virulence factors.^3^ The ability of *Bartonella* spp. to avoid phagocytosis, which it initially accomplishes by replicating intracellularly, is important to its pathogenesis. Extracellularly, it avoids phagocytosis in part due to weak recognition of *Bartonella* lipopolysaccharide (LPS) by phagocyte TLR4 receptors. Weak recognition of *Bartonella* LPS is posited to be due to the lack of an O-chain polysaccharide and the atypical structure of the lipid A, which contains an acyloxyacyl residue.^3^ The lack of an O-side chain may also decrease complement fixation.^3^ Additional mechanisms employed to avoid phagocytosis may include autoaggregation via the *Bartonella* adhesion A (BadA) outer-membrane protein, which is structurally homologous to *Yersinia* adhesin A (YadA). Bacterial aggregates were visualized via immunofluorescence and silver impregnation in this case (Fig. 2), which may support this immune-avoidance mechanism.

This case provides an example of pyogranulomatous myositis and pancarditis, which have previously been described in cases of feline bartonellosis, with the unique addition of a prominent leiomyositis affecting the smooth muscle of the gastrointestinal tract. Cardiac and skeletal muscle lesions associated with *B. henselae* have previously been described in two cats from North Carolina.^10^ The recognition of this bacteria as a cause of pyogranulomatous myocarditis and endocarditis in cats and dogs is becoming more widely recognized. The myotropic pyogranulomatous inflammation provides a striking example of bartonellosis, which importantly could clinically and histologically be mistaken for FIP. Given the sensitivity of FIP IHC and the lack of immunoreactivity within the lesions in this case, concurrent feline coronavirus infection seemed unlikely. Cavitary effusions in this case, attributed to increased vascular permeability associated with the systemic inflammation, provided an additional mimic for FIP. The endotheliotropic nature of *Bartonella* and its effect on the microvasculature likely contributed to the effusions and marked subcutaneous edema.

This case serves as a reminder of the utility of silver impregnation for identification of bacteria, as well as the use of additional ancillary testing such as PCR from fresh-frozen tissue to assess for the presence of pathogen DNA. It also serves as a reminder for the need of employing indirect immunofluorescence or IHC to identify *Bartonella* spp. within lesions, due to the high prevalence of asymptomatic infections within the feline population.
